# Omentoplasty in Surgical Interventions: A Comprehensive Review of Techniques and Outcomes

**DOI:** 10.7759/cureus.66227

**Published:** 2024-08-05

**Authors:** Shruthi Bikkumalla, Suresh R Chandak, Anup A Zade, Srinivasa Reddy, Poosarla Ram Sohan, Akansha Hatewar

**Affiliations:** 1 General Surgery, Jawaharlal Nehru Medical College, Datta Meghe Institute of Higher Education and Research, Wardha, IND

**Keywords:** wound healing, infection control, surgical outcomes, omentum, surgical technique, omentoplasty

## Abstract

Omentoplasty, a surgical technique utilizing the omentum's unique properties, has become a pivotal intervention across various surgical fields. This comprehensive review explores the historical evolution, techniques, applications, outcomes, and complications associated with omentoplasty. With its rich vascular supply, lymphatic tissue, and remarkable immunological properties, the omentum has proven invaluable in enhancing wound healing, controlling infections, and providing mechanical support in complex surgical scenarios. The review delves into the anatomy and physiology of the omentum, elucidating its role in promoting angiogenesis and combating infections. Different omentoplasty techniques, including open, laparoscopic, and robotic-assisted approaches, are compared with regard to indications, procedural steps, and outcomes. The applications of omentoplasty span general surgery, cardiothoracic surgery, neurosurgery, gynecologic surgery, and urologic surgery, highlighting its versatility and broad clinical relevance. Short-term and long-term outcomes of omentoplasty, including postoperative recovery, complication rates, recurrence rates, and quality of life, are thoroughly analyzed. The review addresses common and rare complications, emphasizing prevention and management strategies to optimize patient outcomes. Innovations in surgical techniques, the use of biomaterials, and the potential for synthetic or bioengineered omentum are discussed, underscoring the future directions and research opportunities in this field. By providing a detailed examination of omentoplasty, this review aims to enhance understanding, guide clinical practice, and inspire future research to further improve surgical outcomes and patient care.

## Introduction and background

Omentoplasty is a surgical technique that involves the use of the omentum, a large fold of peritoneum that extends from the stomach and covers the abdominal organs, to support and enhance the healing process in various surgical procedures [1]. This technique leverages the omentum's unique properties, including its rich vascular supply, lymphatic tissue, and ability to adhere to and encapsulate areas of inflammation or infection. The history of omentoplasty dates back to the early 20th century when surgeons first recognized the potential of the omentum in promoting wound healing and controlling infection [2]. Initially, it was employed to manage complex abdominal infections and fistulas. Over the years, its applications have expanded across numerous surgical disciplines due to its versatility and beneficial properties [3].

The significance of omentoplasty in surgical interventions cannot be overstated. Its primary advantages include promoting angiogenesis, fighting infections, and providing a mechanical barrier or support to the surgical site [4]. These properties make it invaluable where enhanced healing is crucial, such as in repairing gastrointestinal perforations, reinforcing anastomoses, and treating chronic infections or fistulas. The omentum’s immunological properties also help reduce postoperative infections, which is particularly beneficial in surgeries involving contaminated fields. Omentoplasty has shown promise in cardiothoracic, neurosurgical, gynecological, and urological procedures, highlighting its broad applicability and vital role in modern surgery [5].

The primary objective of this comprehensive review is to examine the techniques, applications, outcomes, and complications associated with omentoplasty across various surgical fields. By providing an in-depth analysis of existing literature and clinical studies, this review aims to offer valuable insights into the efficacy and safety of omentoplasty, identify areas for improvement, and highlight potential future directions for research and innovation. The scope of this review encompasses a wide range of surgical disciplines, including general surgery, cardiothoracic surgery, neurosurgery, gynecologic surgery, and urologic surgery. Additionally, it explores advancements in surgical techniques, the integration of new technologies, and the potential for synthetic or bioengineered omentum substitutes. This comprehensive review aims to enhance understanding of omentoplasty and its role in improving surgical outcomes.

## Review

Anatomy and physiology of the omentum

Structure and Functions of the Omentum

The omentum is a complex anatomical structure located within the abdominal cavity, consisting of two main parts: the greater and lesser omenta. The greater omentum is a large, apron-like fold of peritoneum that extends from the greater curvature of the stomach over the intestines. This four-layered structure comprises two layers of peritoneum folded upon itself. It is highly vascular, featuring an anastomotic arcade of blood vessels that enhances blood flow in response to inflammation. Its smooth and slippery surface allows it to move freely within the abdominal cavity [6]. Conversely, the lesser omentum is a smaller fold of peritoneum connecting the liver to the lesser curvature of the stomach. The hepatogastric and hepatoduodenal ligaments form the anterior surface of the lesser sac [7]. Beyond its anatomical structure, the omentum plays crucial physiological roles. Known as the "immunological policeman" of the abdomen, it contains immune cells such as macrophages and lymphocytes that respond to infection or injury. The omentum can migrate to sites of inflammation and adhere to them, helping to contain the spread of infection. Additionally, it can increase blood flow and promote angiogenesis in response to inflammation, supporting its immune and regenerative functions [8]. The omentum also serves structural and metabolic roles, providing support and coverage for abdominal organs and storing fat deposits, acting as an energy reserve. Moreover, it aids in isolating wounds and infections by wrapping around affected areas, thereby assisting in the body’s healing process [2].

Role of the Omentum in Healing and Immune Response

**Figure 1:**
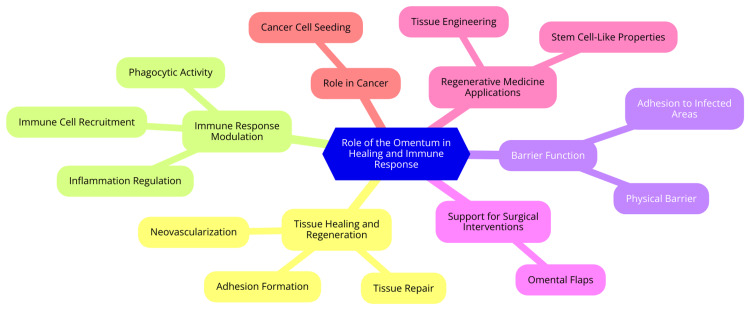
The role of the omentum in healing and immune response. Image Credit: Dr. Shruthi Bikkumalla

The omentum is pivotal in the healing and immune response within the peritoneal cavity. It develops from mesothelial cells and connects to the spleen, stomach, pancreas, and colon. In humans, the omentum is a large, apron-like fold, covering an area of approximately 1500 cm², whereas in mice, it is a relatively small strip [8]. Rich in immune cells such as macrophages and lymphocytes, the omentum features milky spots. These spots function as units that facilitate the rapid influx of inflammatory mediators into the peritoneal cavity in response to injury or infection. Additionally, the omentum contains resident inflammatory and stem cells that play roles in local infection control, wound healing, and tissue regeneration [9]. Its remarkable healing capabilities include its ability to migrate to sites of inflammation and adhere to them, helping to contain the spread of infection. It can enhance blood flow and promote angiogenesis in response to inflammation, supporting its immune and regenerative functions. The omentum contains factors that promote healing and tissue regeneration, making it vital in the body's response to injury and infection [2]. The absence of the omentum, as seen in cases of omentectomy, can lead to increased mortality and morbidity in conditions such as sepsis, underscoring its critical role in peritoneal defense mechanisms. The omentum's ability to move within the peritoneal cavity and sequester areas of inflammation and injury makes it a key player in controlling peritonitis and other abdominal infections [10]. The role of the omentum in healing and immune response is shown in Figure 1.

Techniques of omentoplasty

Open Omentoplasty

Open omentoplasty is a surgical technique that leverages the unique properties of the greater omentum to address various medical conditions. Often referred to as the "policeman of the abdomen," the omentum can wrap around abdominal structures and revascularize areas deprived of blood supply. This technique is employed in both intra-abdominal and extra-abdominal settings to treat various conditions [1]. Intra-abdominally, it has been used for managing hydatid disease of the liver, perforated peptic ulcers, colorectal anastomoses, and surgically treatable pancreatic conditions. Cardiothoracic applications include treating bronchopleural fistulas, poststernotomy mediastinitis, and chest wall reconstruction. In urology, indications for open omentoplasty include pyeloureterostomy, pyelovesicostomy, and omentovesicopexy for neurogenic bladder [1]. Extra-abdominally, it extends to vascular and reconstructive procedures, such as revascularization in peripheral vascular disease and pharyngoesophageal reconstruction, as well as treating filarial lymphedema [11]. The technique involves several key steps. Initially, the transverse colon and stomach are delivered out of the abdomen. The greater omentum is then detached from the transverse colon, and the arterial pattern of the omental vessels is carefully studied. It is subsequently detached from the greater curvature of the stomach, preserving the gastroepiploic arterial arch in the omental pedicle graft. The omental pedicle is lengthened by dividing it according to the anatomical pattern of the vessels. Finally, the omental pedicle is withdrawn from the abdomen through a suprainguinal incision, tunneled subcutaneously to the upper thigh, and fixed to the gastrocnemius muscle [12]. While open omentoplasty is widely utilized across various surgical interventions, it is not without potential complications, including ileus, infection, abscess formation, intestinal obstruction, and total necrosis of the omental flap. Additionally, herniation of abdominal contents through the tunnel created for the omental pedicle can occur [13].

Laparoscopic Omentoplasty

Laparoscopic omentoplasty is a minimally invasive surgical technique that utilizes the greater omentum to fill pelvic defects and reduce the risk of complications in certain digestive, urological, and gynecological surgeries. This procedure has proven particularly beneficial in cases of terminal colostomy, where it helps to address the pelvic defect created during surgery [14]. The procedure begins by detaching the greater omentum from the transverse colon and stomach, ensuring the gastroepiploic arterial arch is retained in the omental pedicle graft. The omental pedicle is then lengthened by dividing it according to the anatomical pattern of the vessels. Next, the omental pedicle is withdrawn from the abdomen through a suprainguinal incision and tunneled subcutaneously to the upper thigh, affixed to the gastrocnemius muscle [15]. Strict hemostasis is maintained throughout the procedure, and the arterial pattern of the omental vessels is carefully examined to select the appropriate feeding vessel for the pedicled graft. It is crucial to avoid tension on the omental pedicle and to keep it moist during the operation. Transverse incisions are made over the medial aspect of the thigh and leg, and a subfascial tunnel is created to advance the omental pedicle [16]. Laparoscopic omentoplasty is recommended for digestive surgeons as it offers a minimally invasive method for filling pelvic defects and minimizing complications in various surgical procedures. The technique requires careful mobilization of the omentum while preserving its blood supply and accurately tunneling it to the desired location, thereby ensuring a successful outcome for the patient [17].

Robotic-Assisted Omentoplasty

Robotic-assisted omentoplasty utilizes the da Vinci Surgical System to perform omentoplasty procedures, offering several advantages over traditional open or laparoscopic techniques, including improved visualization, enhanced dexterity, and reduced surgical trauma [18]. The indications for robotic-assisted omentoplasty are similar to those for conventional omentoplasty. It can be used to fill dead space in the pelvis after extralevator abdominoperineal resection or extended excision for locally advanced or recurrent rectal cancer. Additionally, the omentum can cover defects, augment arterial or portal venous circulation, absorb effusions, or increase lymphatic drainage. Intra-abdominal applications include hydatid disease of the liver, perforated peptic ulcers, colorectal anastomoses, and surgically treatable pancreatic conditions. Extra-abdominal applications encompass revascularization in peripheral vascular disease, bronchopleural fistulas, poststernotomy mediastinitis, chest wall reconstruction, pharyngoesophageal reconstruction, and filarial lymphedema [19]. The robotic-assisted surgical technique involves several key steps. First, the omentum is retracted to the supramesocolic compartment, and the small bowel is retracted to visualize the surgical field. Next, the greater omentum is detached from the transverse colon while preserving the arterial pattern of the omental vessels. The omentum is then detached from the greater curvature of the stomach, retaining the gastroepiploic arterial arch in the omental pedicle graft. The omental pedicle is lengthened by dividing it according to the anatomical pattern of the vessels. Finally, the omental pedicle is withdrawn from the abdomen through a suprainguinal incision and tunneled subcutaneously to the desired location [20]. Robotic-assisted omentoplasty offers several potential benefits. It can help avoid a large pelvic dead space containing serum and blood, which can promote bacterial growth. The omentum also possesses immune regulatory and tissue regenerative properties. An omental buffer between the perineal wound and the small bowel or bladder can also prevent fistula formation and mechanical ileus in a narrow pelvis [21]. However, it is important to note that the effectiveness of omentoplasty, including robotic-assisted techniques, still needs to be debated due to a lack of convincing data and research. Potential downsides include additional surgical time and the risk of omental flap necrosis. Further studies are needed to fully evaluate the outcomes and cost-effectiveness of robotic-assisted omentoplasty compared to traditional techniques [4].

Comparative Analysis of Different Techniques

Omentoplasty, a surgical technique involving the greater omentum, has various applications in intra-abdominal and extra-abdominal settings. One commonly used approach is the omental transposition technique. In this method, the transverse colon and stomach are delivered out of the abdomen, and the greater omentum is detached from the transverse colon while retaining the gastroepiploic arterial arch in the omental pedicle graft. The omental pedicle is then lengthened by dividing it according to the anatomical pattern of the vessels. It is then withdrawn from the abdomen through a suprainguinal incision, tunneled subcutaneously to the upper thigh, and finally fixed to the gastrocnemius muscle [22]. Another technique specifically used in colorectal anastomosis is the colorectal anastomotic omentoplasty technique. This method is faster and easier than omental wrapping and suture fixation. It involves mobilizing the omentum and placing it between the bowel anastomosis and the pelvis to fill the dead space. A systematic review and meta-analysis have found that omentoplasty following gastrointestinal anastomosis may decrease anastomotic leakage without affecting the rate of other complications compared to partial omentectomy [23]. To optimize the omentoplasty procedure, intraoperative fluorescence angiography (FA) can be used to assess perfusion and guide the resection of ischemic omentum. In a cohort study, the use of FA led to additional resection of ischemic omentum in 78% of cases, with a median resection of 37 grams. Furthermore, FA changed management in 78% of cases and created a gluteal turnover flap in one case due to insufficient omental bulk after resection of ischemic tissue [24].

Applications of omentoplasty in various surgical fields

General Surgery

General surgery is a broad specialty that encompasses a wide range of surgical procedures, including hernia repair and gastrointestinal surgery. Hernia repair is a common procedure addressing a weakness or hole in the abdominal wall, allowing internal organs or tissues to protrude. Types of hernias include inguinal, femoral, umbilical, and incisional hernias. The surgical approach for hernia repair may vary depending on the type and size of the hernia, as well as the patient's characteristics [25]. Gastrointestinal surgery is another major area of general surgery, focusing on treating conditions affecting the digestive system. This includes procedures such as appendectomy (removal of the appendix), cholecystectomy (removal of the gallbladder), and various types of bowel resections. Gastrointestinal surgery may be necessary to treat conditions such as appendicitis, gallstones, intestinal blockages, or colorectal cancer. The specific surgical approach depends on the location and severity of the condition, as well as the patient's overall health status [26]. General surgery also encompasses a range of other procedures, such as breast surgery, endocrine surgery, and trauma surgery. Breast surgery may involve the treatment of breast cancer, breast reduction, or breast reconstruction. Endocrine surgery focuses on the surgical treatment of conditions affecting the endocrine glands, such as the thyroid, parathyroid, and adrenal glands. Trauma surgery is concerned with the surgical management of injuries resulting from accidents, falls, or other traumatic events [27]. Recent advances in surgical techniques and technologies have led to the development of minimally invasive surgical approaches, such as laparoscopic and robotic-assisted surgery. These techniques often result in smaller incisions, reduced postoperative pain, shorter hospital stays, and faster patient recovery. However, the surgeon determines the specific surgical approach based on the patient's individual needs and the complexity of the procedure [28].

Cardiothoracic Surgery

Cardiothoracic surgery is a medical specialty that involves surgical procedures on the organs within the thoracic cavity, including the heart, lungs, esophagus, and other mediastinal structures. Cardiothoracic surgeons are highly trained specialists who perform complex surgical interventions to treat cardiovascular and thoracic conditions [29]. Cardiothoracic surgeons perform various cardiac procedures, such as coronary artery bypass grafting (CABG), which uses a blood vessel from another part of the body to bypass a blocked or narrowed coronary artery, thereby improving blood flow to the heart. They also perform heart valve repair or replacement to treat damaged or diseased heart valves, including the aortic, mitral, tricuspid, or pulmonary valves. Aortic aneurysm repair is another common cardiac procedure to treat an enlargement or bulge in the aorta, the main artery that carries blood from the heart to the rest of the body. Cardiothoracic surgeons may also implant cardiac assist devices, such as ventricular assist devices (VADs) or pacemakers, to enhance the heart's pumping efficiency. In severe cases of heart failure, they may perform heart transplantation to replace the patient's diseased heart with a healthy donor heart [30]. In addition to cardiac procedures, cardiothoracic surgeons perform various thoracic procedures. These include lung cancer surgery involving lobectomy, segmentectomy, and video-assisted thoracoscopic surgery (VATS) to remove cancerous lung tissue. They also treat esophageal conditions, such as cancer, hernias, and achalasia, through procedures like esophagectomy and Nissen fundoplication. Tracheal surgery, such as tracheal resection or other airway reconstruction, may be performed to address conditions like tracheal stenosis or tracheal tumors. Cardiothoracic surgeons can also address chest wall deformities, such as pectus excavatum, through procedures like the Ravitch technique [31].

Neurosurgery

Omentoplasty has found applications in various neurosurgical procedures as well. In the context of spinal cord injury, omentoplasty has been used to support neuron regeneration across a freshly transected spinal cord in experimental models. There are published reports indicating that omental transplantation in acute and chronic spinal cord injuries can result in unexpected recovery of limb function [32]. In intracranial procedures, omentoplasty has been utilized in patients with temporal lobe epilepsy, where omental tissue is positioned directly on the epileptic focus, leading to a reduction in seizures. Additionally, intracranial omentum transplantation has shown benefits for patients with cerebrovascular moyamoya disease, a rare progressive cerebrovascular disorder. In these cases, omental transplantation has improved neovascularization and significant clinical neurological improvements [2]. Omental microsurgical free grafts have also been transposed to the subarachnoid space to attempt the closure of cerebrospinal fluid (CSF) fistulas. Furthermore, transposing the omental flap onto the brain surface can generate neoangiogenesis, enhancing the healing of brain injuries by increasing blood flow and oxygen levels. In these cases, the neurotransmitters and neurotrophic factors in the omentum also restore neurological functions [32].

Gynecologic Surgery

Omentoplasty has been effectively utilized in gynecologic and pelvic surgeries to address various complications and improve patient outcomes. In gynecologic surgery, it can cover defects, abscesses, and fistulas that may occur during gynecologic or urological procedures. Additionally, omentoplasty prevents radiation enteritis and facilitates hemostasis during pelvic radiation therapy [33]. The laparoscopic approach to omentoplasty has been proposed as a means to reduce perineal complications associated with pelvic surgeries. This minimally invasive technique involves using a laparoscope to perform the omentoplasty, potentially offering benefits over traditional open methods [34]. The omentum's highly vascularized and thromboplastin-rich nature makes it an excellent material for managing difficult abdominal or pelvic abscesses and promoting hemostasis. Furthermore, the omentum has a trophic effect on surrounding tissues, is beneficial for reconstruction procedures, and can elevate the small intestines out of the true pelvis, facilitating high-dose brachytherapy with reduced risk of radiation enteritis [2]. A specific application of omentoplasty in gynecologic and pelvic surgeries is during pelvic radiation therapy. In this context, omentoplasty creates a safe pelvic radiation field by covering large operating fields instead of reperitonealizing them, which helps prevent radiation enteritis [33]. Clinical experience with pedicled omentoplasty in gynecology has been favorable, with the technique being straightforward and adding only about 20-30 minutes to the operating time. It has been successfully employed in 48 omentoplasty procedures within gynecology, yielding positive outcomes [33].

Urologic Surgery

Bladder surgeries are pivotal in diagnosing and treating various bladder conditions. Cystoscopy is a commonly used procedure that involves inserting a thin, flexible tube with a camera (cystoscope) through the urethra to examine the bladder lining, serving both diagnostic and therapeutic purposes. Bladder tumor resection (TURBT) is a surgical method used to remove bladder tumors via cystoscopy, and it is a key treatment for bladder cancer. Robotic radical cystectomy, which involves the complete removal of the bladder, is another procedure frequently employed for bladder cancer management. Additionally, Botox injections into the bladder can address symptoms of overactive bladder, while bladder stone removal is performed to extract stones from the bladder surgically [35]. Urethral surgeries are focused on repairing and reconstructing the urethra. Urethroplasty is performed to treat urethral strictures or scarring to restore normal urinary function. Bladder neck incision (BNI) involves an incision at the bladder neck to treat stenosis and improve urine flow. Urethrotomy, the surgical incision of the urethra, is another technique used to manage urethral strictures. Various penile procedures, such as penile plication, penile implants, and circumcision, are also performed to address conditions affecting the penis [36]. The primary objectives of these bladder and urethral surgeries are to diagnose, treat, and manage a broad spectrum of urological conditions that can impair urinary function. Achieving favorable outcomes and minimizing complications depend on careful patient selection and meticulous surgical techniques [37].

Outcomes and efficacy of omentoplasty

Short-Term Outcomes

Omentoplasty has been demonstrated to significantly enhance postoperative recovery across various surgical settings. This technique is associated with a lower incidence of overall postoperative complications, particularly in gastrointestinal and liver surgeries. Additionally, omentoplasty reduces the risk of postoperative infections in thoracic and liver surgeries. In patients with a BMI over 25 undergoing esophageal and GI surgeries, omentoplasty notably lowers the risk of overall complications compared to those with a normal BMI. These observations suggest that omentoplasty can improve postoperative outcomes by reducing complications and enhancing recovery [4]. A systematic review and meta-analysis further confirm the effectiveness of omentoplasty in lowering complication rates across different surgical contexts. For example, omentoplasty was linked to a reduced risk of overall complications in gastrointestinal surgery (RR 0.53; 95% CI: 0.39-0.72) and liver surgery (RR 0.54; 95% CI: 0.39-0.74). It also decreased the risk of postoperative infections in thoracic surgery (RR 0.38; 95% CI: 0.18-0.78) and liver surgery (RR 0.39; 95% CI: 0.29-0.52). These findings underscore the beneficial impact of omentoplasty on reducing complications and improving patient outcomes [4]. However, the effectiveness of omentoplasty may vary depending on the specific surgical procedure and patient characteristics, such as BMI. For instance, in patients undergoing esophageal and gastrointestinal surgeries with a BMI greater than 25, omentoplasty was significantly associated with a decreased risk of overall complications compared to those with a normal BMI (RR 0.28; 95% CI: 0.23-0.34). On the other hand, in pelvi-perineal surgeries, no significant differences in complication rates were observed, although there was an increased risk of infection in patients with a BMI between 25 and 29.9 kg/m² (RR 1.25; 95% CI: 1.04-1.50) and a reduced risk of anastomotic leakage in patients over 60 years old (RR 0.59; 95% CI: 0.39-0.91). These findings highlight the importance of considering patient-specific factors when assessing the efficacy of omentoplasty [4].

Long-Term Outcomes

The long-term outcomes of omentoplasty have been studied across various surgical contexts, with particular attention to recurrence rates and quality of life. In managing remnant liver after hepatectomy, omentoplasty alone demonstrated the lowest recurrence rate of 3.2%, compared to other techniques, such as omentoplasty combined with external drainage, capitonnage, or introflexion. This suggests that omentoplasty can effectively minimize recurrence risk in specific surgical settings [38]. However, the impact of omentoplasty quality on long-term outcomes needs to be clarified. A study evaluating the quality of omentoplasty using CT imaging found that insufficient omentoplasty was associated with significantly higher rates of delayed healing at six months (46% vs. 14%) and chronic perineal sinus at 12 months (31% vs. 3%) compared to no omentoplasty. This indicates that the quality of the omentoplasty procedure can influence long-term outcomes. Nevertheless, the same study did not find a significant association between adequate omentoplasty and reduced delayed healing in multivariate analysis, suggesting that other factors may also contribute to these outcomes [39]. Regarding quality of life, omentoplasty has shown significant benefits for patients with advanced lymphedema. A study reported that the minimally invasive free vascularized omental lymphatic flap resulted in subjective improvements for 83% of patients at a mean follow-up of 14 months. Additionally, this technique was associated with a 22% mean volumetric improvement in lymphedema. These results highlight the value of omentoplasty in enhancing the quality of life for patients with advanced lymphedema. While omentoplasty has demonstrated promise in reducing recurrence rates and improving quality of life in certain surgical scenarios, further research is needed to fully assess its long-term efficacy and outcomes [40].

Comparative Outcomes With and Without Omentoplasty

The systematic review and meta-analysis revealed that omentoplasty is associated with a significantly lower risk of overall postoperative complications, particularly in gastrointestinal (RR 0.53, 95% CI 0.39-0.72) and liver surgeries (RR 0.54, 95% CI 0.39-0.74), compared to no omentoplasty. This complication reduction was also observed in thoracic surgery, where omentoplasty decreased the risk of postoperative infection (RR 0.38, 95% CI 0.18-0.78). These findings suggest that omentoplasty can substantially improve postoperative outcomes across various surgical settings [4]. In patients undergoing esophageal and gastrointestinal surgeries with a BMI greater than 25, omentoplasty was significantly associated with a reduced risk of overall complications compared to those with a normal BMI. For example, in esophageal surgery, omentoplasty reduced the risk of overall complications by 11% (RR 0.89, 95% CI 0.80-0.99), and in gastrointestinal surgery, the risk was reduced by 72% (RR 0.28, 95% CI 0.23-0.34). This indicates that omentoplasty may be particularly beneficial for patients with higher BMI undergoing these procedures [4]. In contrast, the impact of omentoplasty on outcomes in pelvi-perineal surgeries appears more variable. While no significant differences in outcomes were observed in most cases, there was an increased risk of infection in patients with a BMI between 25-29.9 kg/m² (RR 1.25, 95% CI 1.04-1.50). However, omentoplasty did reduce the risk of anastomotic leakage in patients over 60 years old (RR 0.59, 95% CI 0.39-0.91). These findings suggest that the benefits of omentoplasty in pelvic-perineal surgeries may be more nuanced and dependent on specific patient characteristics [4].

Complications and management

Common Complications

Omentoplasty utilizes a portion of the greater omentum to cover or fill defects, augment blood circulation, absorb effusions, or enhance lymphatic drainage in various surgical contexts. Despite its benefits, omentoplasty can be associated with several complications, particularly infections. Infections are notably concerning, especially with extra-abdominal applications of omentoplasty. For example, one study reported infections in 7 patients who underwent extra-abdominal omentoplasty. Abdominal wall infection was the most common complication, affecting nine patients, particularly those who had pedicled reconstruction of contaminated or infected thoracic wounds. Prompt recognition and management of infections are essential to prevent further complications [1]. Another common complication of omentoplasty is hemorrhage. Delayed splenic rupture and gastrointestinal hemorrhage have been reported as potentially life-threatening complications. Careful surgical technique and effective hemostasis during the procedure are crucial to minimize the risk of bleeding. Additionally, adhesions and hernias are frequent complications associated with omentoplasty. Incisional hernia, requiring operative correction, occurred in 7 patients in one study. Hernia and fascial dehiscence were predominant donor-site complications. Relative contraindications for omentoplasty include insufficient omental length due to prior infections or surgeries, which may increase the risk of adhesions and hernias. Therefore, careful patient selection and meticulous surgical technique are critical to minimize these complications [41].

Rare Complications

Necrosis is a rare but serious complication associated with omentoplasty, occurring in 2-4% of cases. This complication can arise from unrecognized intraoperative ischemia of the omentum, leading to postoperative necrosis and potentially resulting in secondary infections. To mitigate this risk, careful intraoperative assessment of omental perfusion, such as through FA, can help identify and resect ischemic parts of the omentum, thereby preventing necrosis [24]. Fistula formation is another rare but notable complication linked to omentoplasty. An unusual case involved the herniation of the transverse colon through the tunnel created for transposing the omentum to the thigh, leading to a fistula between the transposed omentum and the transverse colon. Other rare instances of gastrointestinal fistula formation related to omentoplasty have been reported, including a case where a gastric carcinoid tumor metastasized to the head and neck region following omental harvest for pharyngeal reconstruction [42].

Strategies for Prevention and Management

Careful patient selection is essential when considering omentoplasty for surgical interventions. This technique should be avoided in patients with advanced intra-abdominal malignancies or those with insufficient omental length or poor-quality blood vessels. Such conditions increase the risk of complications, including necrosis, infection, and herniation [43]. Meticulous surgical technique is vital to minimizing complications associated with omentoplasty. Key steps include preserving the right gastroepiploic artery and ensuring adequate blood supply to the omental flap to prevent tissue necrosis. Omentoplasty has demonstrated efficacy in reducing postoperative complications such as anastomotic leakage, surgical site infections, and bile fistulas across various surgical settings, highlighting the importance of proper execution [44]. When complications do arise, prompt recognition and management are critical. For instance, endoscopic stenting has been employed to address anastomotic leaks after esophageal surgery, although its effectiveness in cervical esophagogastrostomy remains uncertain. The pedicled omental flap can help seal microscopic leaks and protect the anastomosis from gastric fluid spread, potentially reducing leakage-associated mortality. Effective monitoring and early intervention are crucial for addressing complications such as ileus, necrosis, infection, and hernias, thereby optimizing patient outcomes [45].

Innovations and future directions

Advances in Surgical Techniques

Minimally invasive surgery (MIS) has transformed the surgical landscape by offering smaller incisions, reduced scarring, and quicker recovery times compared to traditional open surgeries. Laparoscopy, a cornerstone of MIS, enables surgeons to perform complex abdominal procedures through small keyhole incisions, leveraging specialized instruments and cameras for enhanced visualization and control [46]. The advent of robotic systems, such as the da Vinci Surgical System, has further advanced minimally invasive techniques. These systems provide 3D visualization, magnification, and superior maneuverability, facilitating delicate and precise movements within confined spaces. Robotic-assisted surgeries have improved outcomes across various procedures, including urological, gynecological, and thoracic surgeries [47]. Augmented reality (AR) and virtual reality (VR) technologies revolutionize surgical training and planning. Surgeons can now utilize virtual simulations to practice procedures, enhancing their skills and confidence. AR-assisted surgeries integrate real-time patient data and vital statistics directly into the surgeon's field of view, enhancing decision-making and reducing errors. These technologies also create immersive training environments, allowing surgeons to practice complex procedures safely and effectively [48].

3D printing has introduced the capability to produce patient-specific implants and anatomical models for pre-surgical planning. Accurate replicas of complex anatomical structures can be created to aid in visualization and practice before actual surgery. This technology is particularly beneficial in orthopedic and craniofacial surgeries, where precise reconstruction and alignment are crucial. Additionally, 3D printing allows for the creation of custom implants tailored to individual patients, improving the fit and functionality of prosthetic devices [49]. Nanotechnology is being explored for its potential in targeted drug delivery, imaging, and tissue engineering. Nano-sized particles enable precise drug delivery, reducing side effects and enhancing therapeutic efficacy. Nanoparticles also offer high-resolution imaging capabilities for tissues and organs, and in tissue engineering, nanomaterials can create scaffolds that replicate the natural cellular environment, fostering tissue regeneration and repair [50]. Artificial Intelligence (AI) is increasingly utilized to analyze patient data, aiding surgeons in making informed decisions. Machine learning algorithms assist with preoperative planning, risk assessment, and real-time decision support during surgeries. AI can also identify potential complications and provide personalized treatment strategies. As technology advances, these innovations are expected to further enhance surgical outcomes and patient care [51].

Use of Biomaterials and Synthetic Omentum

Using biomaterials and synthetic omentum in surgical interventions represents a significant area of innovation and future direction for omentoplasty. These advancements can potentially enhance the regenerative and healing properties of the omentum, offering new possibilities for various surgical applications [2]. One notable example is the decellularized omentum scaffold. This scaffold, developed for use with mesenchymal stem cells and platelet-rich plasma (PRP), has shown promise in healing critical-sized bone defects. It provides a supportive matrix that promotes tissue regeneration and bone formation by facilitating cell growth and differentiation [52]. Another innovative biomaterial is the omentum extracellular matrix-silk fibroin hydroscaffold. Created using decellularized omentum tissue-derived extracellular matrix (ECM) and silk fibroin (SF), this biomimetic hydro scaffold enhances tissue regeneration and vascularization, making it suitable for various surgical applications [53]. In synthetic omentum, synthetic meshes are employed in abdominal wall hernia repair. These meshes, which include permanent and absorbable materials, act as scaffolds for tissue ingrowth and help reduce adhesion formation by creating a neoperitoneum. However, the long-term effectiveness and potential for adhesion formation with synthetic meshes are still under investigation [54].

Looking ahead, several areas of research and innovation could further advance the field of omentoplasty. Minimally invasive techniques for omentoplasty could reduce surgical trauma and improve patient outcomes. Advances in tissue engineering and regenerative medicine may enhance the revascularization and regenerative properties of the omentum, potentially broadening its applications [24]. Additionally, leveraging the omentum's ability to adhere to surrounding structures and develop vascular connections could enable targeted drug delivery to specific organs or tissues, potentially improving treatment efficacy. Personalized approaches that consider patient-specific factors could optimize the application of omentoplasty and lead to more tailored surgical interventions [55]. Exploring the synergistic effects of omentoplasty combined with other surgical or non-surgical interventions, such as tissue engineering or pharmacological treatments, could expand its clinical utility. Finally, developing reliable biomarkers or predictive models to identify patients most likely to benefit from omentoplasty could improve patient selection and outcomes [4].

Role of Omentoplasty in Regenerative Medicine

Omentoplasty, a surgical technique utilizing part of the greater omentum to cover or fill a defect, has demonstrated significant potential in regenerative medicine. The omentum's unique biological properties make it a valuable asset in regenerative surgery [1]. It contains various cell clusters with stem cell-like qualities, including mesenchymal stem cells (MSCs) that can differentiate into multiple cell types, such as bone, fat, cartilage, cardiomyocytes, lung epithelial cells, hepatocytes, neurons, and pancreatic islets [56]. Additionally, the omentum supports neovascularization, hemostasis, tissue healing, and regeneration. Its capacity to enhance healing has been utilized for over a century, although the precise mechanisms remain partially understood [2]. Omentoplasty has found applications across diverse fields of regenerative surgery, including orthopedic, wound healing, vascular, cardiothoracic, urologic, and reconstructive procedures. It has been used to treat septic lesions of costal cartilage and sternum, prevent adhesions following tenolysis, cover nerve injuries, promote healing of full-thickness necrotic infections, facilitate revascularization in peripheral vascular disease, and address bronchopleural fistula, poststernotomy mediastinitis, and various urologic and reconstructive conditions such as pyeloureterostomy, pyelovesicostomy, omentovesicopexy, pharyngoesophageal reconstruction, and filarial lymphedema [57]. Despite its promise, further research is needed to understand the mechanisms of omentoplasty and optimize its applications fully. Innovations such as minimally invasive techniques, advancements in tissue engineering, and targeted drug delivery could further enhance the clinical utility of this versatile surgical tool [58].

## Conclusions

Omentoplasty has emerged as a versatile and invaluable technique in the surgical armamentarium, offering significant benefits across various fields of surgery. Leveraging the omentum’s unique properties, such as its rich vascular supply, immunological functions, and ability to promote tissue healing, surgeons can enhance outcomes of complex procedures, manage infections more effectively, and reduce postoperative complications. This comprehensive review has highlighted the broad applications of omentoplasty, from general and GI surgery to cardiothoracic, neurosurgical, gynecologic, and urologic interventions. It has also underscored advancements in surgical techniques, the promising potential of synthetic and bioengineered alternatives, and the directions for future research and clinical practice. As surgical approaches continue to evolve, the role of omentoplasty in improving patient outcomes remains pivotal, demonstrating its enduring significance in modern surgery.
